# Octreotide Attenuates Acute Kidney Injury after Hepatic Ischemia and Reperfusion by Enhancing Autophagy

**DOI:** 10.1038/srep42701

**Published:** 2017-02-16

**Authors:** Huiping Sun, Shuangfa Zou, Keith A. Candiotti, Yanhua Peng, Qinya Zhang, Weiqiang Xiao, Yiyun Wen, Jiao wu, Jinfeng Yang

**Affiliations:** 1 Department of Anesthesiology, Hunan Cancer Hospital, The Affiliated Cancer Hospital of Xiangya School of Medicine, Central South University, Changsha 410013, Hunan, China; 2 Department of Anesthesiology, Perioperative Medicine and Pain Management, University of Miami-Miller School of Medicine, Miami, FL 33136, USA

## Abstract

Octreotide exerts a protective effect in hepatic ischemia-reperfusion (HIR) injury. However, whether octreotide preconditioning could also reduce acute kidney injury (AKI) after HIR is unknown. This study was designed to investigate the role of octreotide in AKI after HIR. Male Sprague-Dawley rats were pretreated with octreotide or octreotide combined with 3-methyladenine (autophagy inhibitor, 3MA). Plasma creatinine, inflammation markers (e.g., TNF-α and IL-6 etc.), apoptosis, autophagy and phosphorylation of protein kinase B/mammalian target of rapamycin/p70 ribosomal S6 kinase (Akt/mTOR/p70S6K) in the kidney were measured after 60 minutes of liver ischemia and 24 hours of reperfusion for each rat. Octreotide pretreatment significantly preserved renal function and reduced the severity of renal injury. Moreover, octreotide alleviated inflammation and apoptosis in the kidney after HIR. Additionally, octreotide induced autophagy and autophagy inhibition with 3MA markedly reversed the renoprotective, anti-inflammatory and anti-apoptotic effects of octreotide after HIR. Finally, octreotide abrogated the activation of phosphorylation of Akt, mTOR and p70S6K in the kidney after HIR. Our results indicate that octreotide reduced renal injury after HIR due to its induction of autophagy. The enhancement of autophagy may be potentially linked to the octreotide mediated Akt/mTOR/p70S6K pathway deactivation and reduction of kidney inflammation and apoptosis after HIR.

Hepatic ischemia and reperfusion injury (HIR) often occurs in complex liver surgeries such as major liver resection and liver transplantation^1^. HIR cannot only result in acute liver injury, but also frequently leads to other organ damage including kidney, lung, and heart etc^2^. Clinically, acute kidney injury (AKI) after major liver surgery is extremely common and the development of AKI after liver injury greatly increases patient mortality and morbidity during the perioperative period^3^,^4^. Despite the global attention to this common clinical condition, AKI remains a diagnostic and therapeutic challenge. Therefore, novel therapeutic strategies and pharmacologic interventions that can reduce the incidence or severity of AKI, as a result of HIR, are highly desirable.

Octreotide is an octapeptide that mimics natural somatostatin pharmacologically. Originally, it was used for the treatment of vasoactive intestinal peptide-secreting tumors, growth hormone producing tumors, and pituitary tumors^5^. In addition to its role in the management of neuroendocrine tumors, octreotide also displays efficacy in the treatment of cluster headaches, acute hemorrhage from esophageal varices resulting from liver cirrhosis, malignant bowel obstruction, and idiopathic intracranial hypertension etc^6^,^7^. Octreotide appears to exert its organ protective effects through several mechanisms^8^,^9^,^10^. A previous study demonstrated that octreotide could protect the liver against HIR in a rabbit model. The protective mechanisms of octreotide appear to be associated with its ability to decrease the levels of endotoxin and the proinflammatory cytokines TNF-α and IL-1β, as well as inhibit hepatocellular apoptosis^11^. However, whether octreotide preconditioning could also reduce renal injury after HIR remains to be investigated.

Autophagy is an evolutionally conserved intracellular degradation pathway responsible for maintaining cellular homeostasis^12^. Basal autophagy plays an important role during development and differentiation. In pathological conditions, autophagy promotes cellular adaptation with cytoprotective effects by eliminating and recycling damaged macromolecules and organelles^12^,^13^. Autophagy has been shown to be induced in response to AKI in both *in vivo* and *in vitro* models. In most studies, inhibition of autophagy worsens renal tubular injury and renal function, supporting the concept that autophagy is renoprotective^14^,^15^,^16^. Therefore, it appears that autophagy is important in AKI and is a potential therapeutic target in the pathogenesis of AKI.

It is well established that the mammalian target of rapamycin/p70 ribosomal S6 kinase (mTOR/p70S6K) pathway is an important regulator of autophagy. Pharmacological inhibition of mTOR can upregulate autophagy^17^ and is positively regulated by the PI3K/Akt signaling pathway^18^. The somatostatin receptor type 2 (Sst2), which octreotide targets, was shown to deactivate the PI3K pathway by inhibiting p85 tyrosine phosphorylation and decreasing Akt phosphorylation^19^. Based on these findings, we hypothesized that octreotide-induced autophagy in the renal proximal tubules will lessen renal tubular injury and preserve renal function after HIR by inhibiting the PI3K/Akt/mTOR/p70S6K signaling pathway. In the present study, we examined the pharmacological function and potential mechanisms of octreotide in AKI after HIR in a rat model.

## Results

### Octreotide protects against kidney injury after HIR

**Figure 1:**
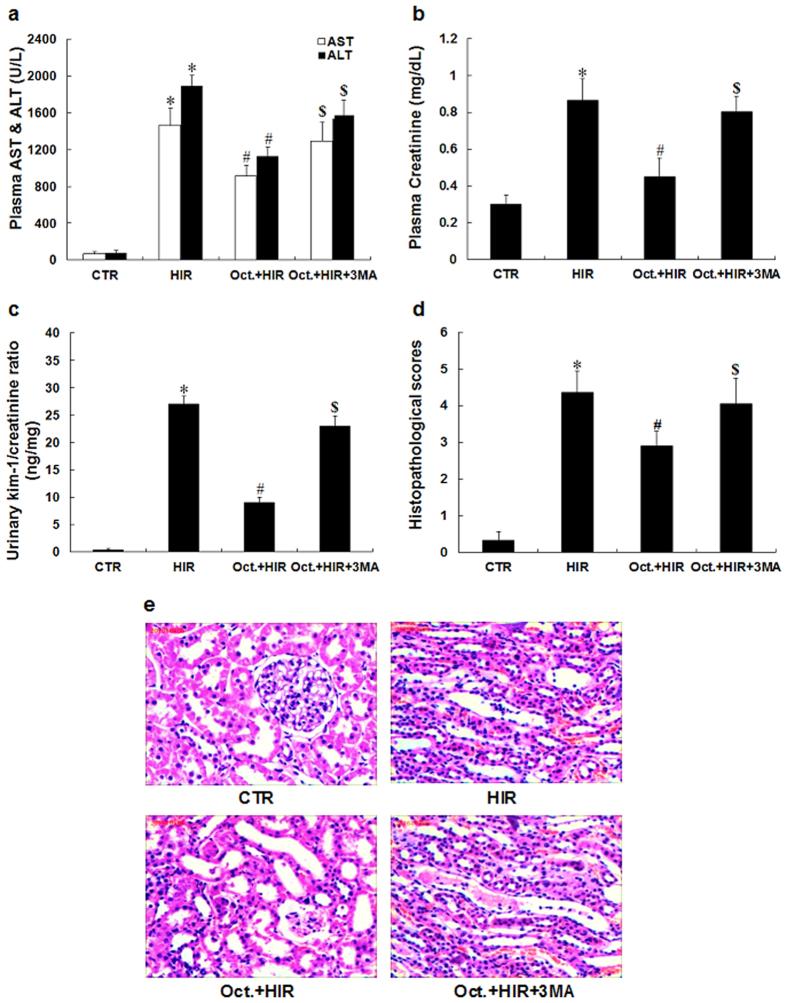
Degree of AKI after HIR. (**a**–**c**) Plasma AST/ALT, creatinine and urinary kim/creatinine ratio were measured after 60 minutes of liver ischemia and 24 hours of reperfusion for each rat. (**d**) Histopathological scoring of kidney injury was performed. (**e**) Representative hematoxylin and eosin-stained photomicrographs of kidney sections from rats that were subjected to a control-operation, HIR, Oct. + HIR, and Oct. + HIR+3MA after 60 min. of liver ischemia and 24 hrs of reperfusion (200× magnification, Bar = 200 μm) are shown. Ten rats were included in each study group. The results are presented as mean ± S.D. *P < 0.05 HIR vs. CTR group, ^#^P < 0.05 Oct. + HIR νs. HIR group, and ^$^P < 0.05 Oct. + HIR+3MA νs. Oct. + HIR group. AKI, acute kidney injury; CTR, control; HIR, hepatic ischemia-reperfusion; AST, aspartate aminotransferase; ALT, alanine aminotransferase; Oct. + HIR, octreotide pretreatment and hepatic ischemia-reperfusion and Oct. + HIR+3MA, octreotide and 3-methyladenine pretreatment and hepatic ischemia-reperfusion.

Plasma AST and ALT were increased in the rat model of HIR (Fig. 1a). The HIR group demonstrated renal dysfunction when compared to the CTR group as reflected by a significant elevation of serum creatinine (0.86 ± 0.12 mg/dL vs. 0.31 ± 0.05 mg/dL, p < 0.05, Fig. 1b) and urinary kim/creatinine ratio (27.1 ± 2.5 ng/mg vs. 0.4 ± 0.3 ng/mg, p < 0.05, Fig. 1c). In addition, histological alterations noted in the kidneys of HIR rats were characterized by widespread tubular necrosis, loss of the brush border, inflammatory cell infiltration, and tubular dilatation. In contrast, pretreatment with octreotide (Oct. + HIR) attenuated the renal pathological changes when compared to the HIR group with no pretreatment (Fig. 1e). The Oct. + HIR group had lower histology scores compared with the HIR group (2.87 ± 0.41 vs. 4.37 ± 0.57, p < 0.05, Fig. 1d). This data supports the potential protective effect of octreotide in the setting of renal injury following HIR.

### Octreotide reduces kidney inflammation after HIR

**Figure 2:**
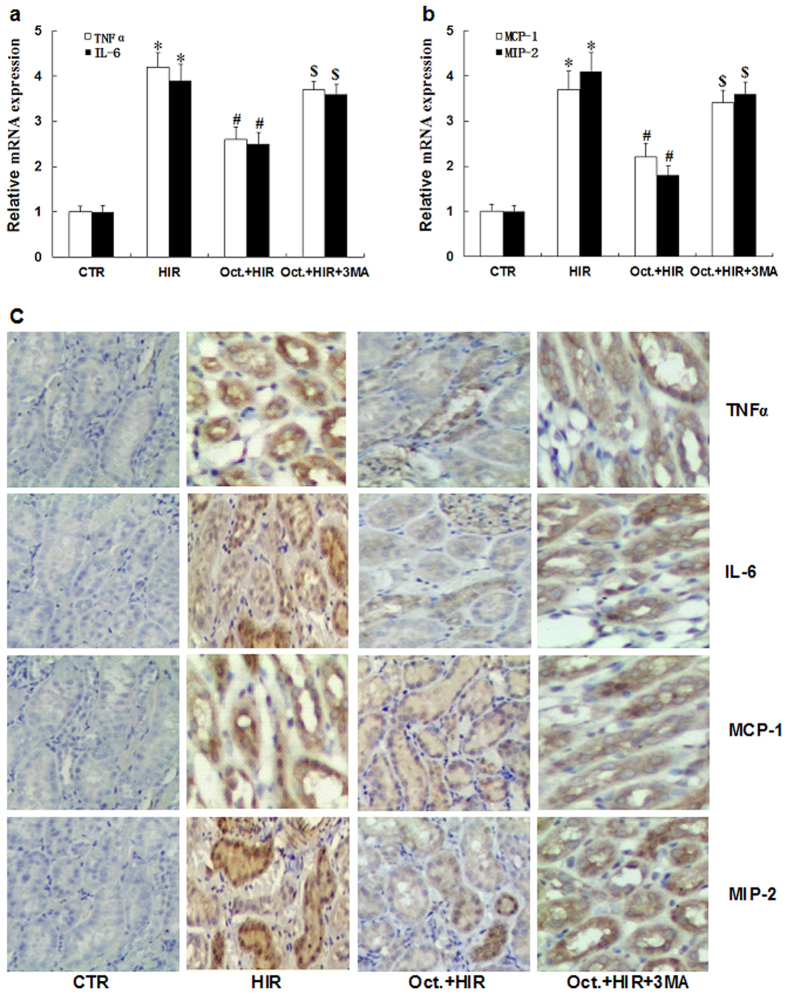
Expression of pro-inflammatory mediators in the kidney after HIR. (**a**,**b**) qRT-PCR analysis of TNFα, IL-6, MCP-1, and MIP-2 mRNA in kidney tissue from rats in the CTR group, HIR, Oct. + HIR, and Oct. + HIR+3MA after 60 minutes of liver ischemia and 24 hours of reperfusion. (**c**) Immunohistochemistry staining results of TNFα, IL-6, MCP-1, and MIP-2 in kidney tissue from rats in the CTR group, HIR, Oct. + HIR, and Oct. + HIR+3MA after 60 min. of liver ischemia and 24 hrs of reperfusion. (200× magnification, Bar = 200 μm). Ten rats were included in each study group. The results are presented as mean ± S.D. *P < 0.05 HIR vs. CTR group, ^#^P < 0.05 Oct. + HIR νs. HIR group, and ^$^P < 0.05 Oct. + HIR+3MA νs. Oct. + HIR group. AKI, acute kidney injury; CTR, control; HIR, hepatic ischemia-reperfusion; Oct. + HIR, octreotide pretreatment and hepatic ischemia-reperfusion and Oct. + HIR+3MA, octreotide and 3-methyladenine pretreatment and hepatic ischemia-reperfusion.

HIR injury has been shown to be associated with a significant increase in the expression of proinflammatory mediators in the kidney^20^. To examine whether octreotide could modulate renal inflammation after HIR, TNFα, IL-6, MCP-1, and MIP-2 expression levels were evaluated with and without octreotide pretreatment by qRT-PCR and immunohistochemistry analysis (HIR vs. Oct.+HIR). As shown in Fig. 2a–c, octreotide significantly suppressed TNFα, IL-6, MCP-1, and MIP-2 mRNA and protein expression in renal tissue from rats subjected to 60 minutes of liver ischemia and 24 hours of reperfusion. This data suggests that octreotide can also inhibit renal inflammation after HIR.

### Octreotide suppresses apoptosis in the kidney after HIR

**Figure 3:**
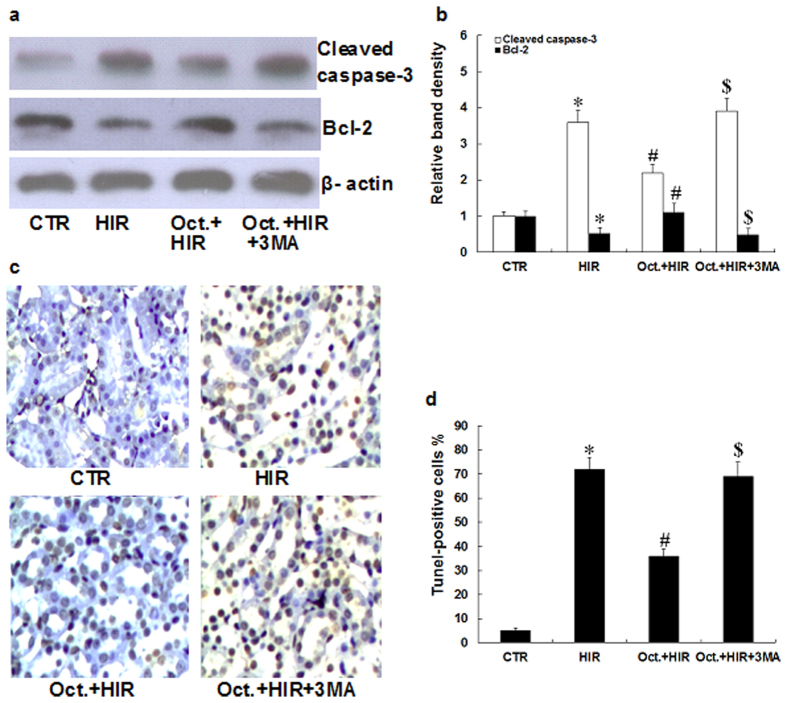
Extent of apoptosis in the kidney after HIR. (**a**,**b**) Representative western blots and quantitative evaluation of cleaved caspase-3 and Bcl-2 expression in kidney tissue from rats in the CTR group, HIR, Oct. + HIR, and Oct. + HIR+3MA after 60 minutes of liver ischemia and 24 hours of reperfusion. (**c**) Tunel staining of kidneys collected after 60 minutes of liver ischemia and 24 hours of reperfusion (200× magnification, Bar = 200 μm). (**d**) Statistical analysis of the percentages of tunel-positive cells in tissue sections. Ten rats were included in each study group. The results are presented as mean ± S.D. *P < 0.05 HIR νs. CTR group, ^#^P < 0.05 Oct. + HIR νs. HIR group, and ^$^P < 0.05 Oct. + HIR+3MA νs. Oct. + HIR group. AKI, acute kidney injury; CTR, control; HIR, hepatic ischemia-reperfusion; Oct. + HIR, octreotide pretreatment and hepatic ischemia-reperfusion and Oct. + HIR+3MA, octreotide and 3-methyladenine pretreatment and hepatic ischemia-reperfusion.

Apoptosis in the kidney is a hallmark of AKI^21^. To examine the effects of octreotide on apoptosis in the kidney, we evaluated its effects on cleaved caspase-3 and Bcl-2 protein, which are often used as indicators of apoptosis. As shown in Fig. 3a,b, HIR induced cleaved caspase-3 protein expression, but inhibited Bcl-2 protein expression in the kidney when compared to the CTR group. However, octreotide pretreatment followed by HIR, increased the expression of Bcl-2 protein, but decreased the expression of cleaved caspase-3 protein when compared to the HIR group. To further assess apoptosis in the kidney, Tunel staining was performed. As shown in Fig. 3c,d, the number of Tunel-positive cells significantly increased in the kidney after HIR. However, the octreotide pretreatment group showed significantly fewer Tunel-positive cells when compared to the HIR group (36.5 ± 3.5 vs. 75.3 ± 5.8, p < 0.05). This data supports the concept that octreotide can suppress apoptosis in the kidney after HIR.

### Octreotide induces autophagy in the kidney after HIR

**Figure 4:**
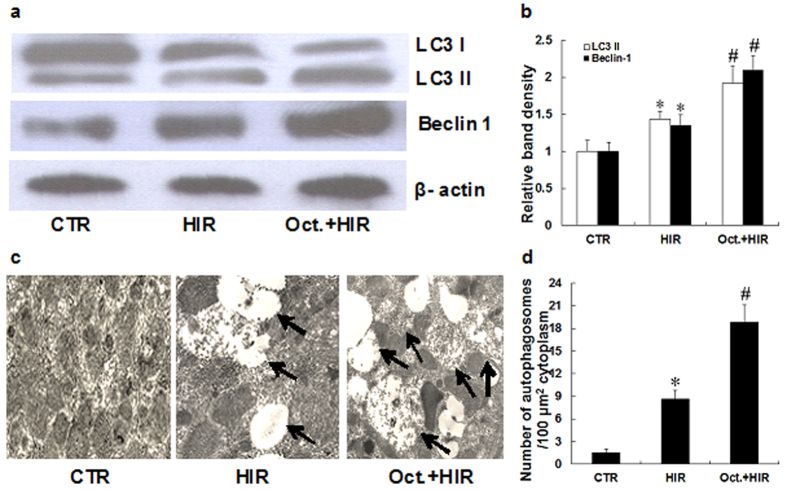
Effect of octreotide on autophagy in the kidney after HIR. (**a**,**b**). Representative western blots and quantitative evaluation of LC3-II and Beclin 1 expression in kidney tissue from rats in the CTR group, HIR, and Oct. + HIR after 60 minutes of liver ischemia and 24 hrs of reperfusion. (**c**) Representative transmission electron microscopy (TEM) images for autophagy ultrastructures. Autophagosomes are indicated by arrows. Bar = 2 μm, (**d**). Statistical analysis of the number of autophagosomes per 100 μm^2^. Ten rats were included in each study group. The results are presented as mean ± S.D. *P < 0.05 HIR vs. CTR group, ^#^P < 0.05 Oct. + HIR νs HIR group. AKI, acute kidney injury; CTR, control; HIR, hepatic ischemia-reperfusion; Oct. + HIR, octreotide pretreatment and hepatic ischemia-reperfusion and Oct. + HIR+3MA, octreotide and 3-methyladenine pretreatment and hepatic ischemia-reperfusion.

Autophagy is important in renal injury and is a potential therapeutic target in the pathogenesis of AKI^22^. To evaluate the effects of octreotide on autophagy in the kidney, we first measured how octreotide impacted the expression of LC3 and beclin-1 protein, which are often used as indicators of autophagy. As shown in Fig. 4a,b, HIR induced LC3 and beclin-1 protein expression slightly in renal tissue when compared to the CTR group. However, octreotide pretreatment followed by HIR, showed a far more significant induction of LC3 and beclin-1 protein expression. To further examine the presence of autophagy, transmission electron microscopy was used to measure the quantity of autophagosomes per unit cytoplasmic area/100 μm^2^. As shown in Fig. 4c,d, the number of autophagosomes per 100 μm^2^ significantly increased in the kidney after HIR. Compared with the HIR group, a greater number of autophagosomes were seen in the Oct. + HIR group (18.8 ± 2.3 vs. 8.6 ± 3.2, p < 0.05). This finding supports the concept that octreotide may induce autophagy in the kidney after HIR.

### Autophagy inhibition reverses the renoprotective effects of octreotide

To further evaluate the role of autophagy and the renoprotective effects of octreotide, we used 3-methyladenine (3MA), an autophagy inhibitor, to further decrease the activity of autophagy in the animals treated with octreotide while undergoing HIR. As shown in Fig. 1b–f, the positive effects of octreotide on serum creatinine levels, urinary kim/creatinine ratio and the renal pathological changes were significantly reversed by the addition of 3MA. Moreover, 3MA abrogated the inhibitory action of octreotide on proinflammatory mediator expression (TNFα, IL-6, MCP-1, and MIP-2) (Fig. 2a–c) and apoptosis (Fig. 3a–d) in the kidney after HIR. This data appears to further support that autophagy is a key mediator in the renoprotective effects of octreotide after HIR.

#### Octreotide suppresses Akt/mTOR/p70S6K signaling pathway in the kidney after HIR

**Figure 5:**
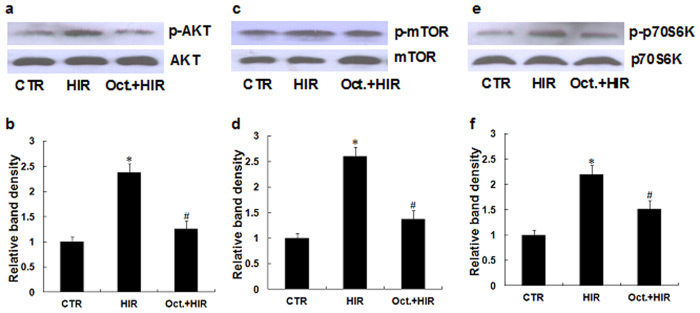
Effect of octreotide on Akt/mTOR/p70S6K signaling pathway in the kidney after HIR. The protein expression of p-Akt (Ser473)/Akt, p-mTOR (Ser2448)/mTOR and p-p70S6K (Ser423)/p70S6K were examined using western blotting analyses. (**a**,**b**) Representative western blots and quantitative evaluation of p-AKT expression in kidney tissue from rats in the CTR group, HIR, and Oct. + HIR after 60 minutes of liver ischemia and 24 hrs of reperfusion. (**c**,**d**) Representative western blots and quantitative evaluation of p-mTOR expression in kidney tissues from the rats subjected to control-operated, HIR, and Oct. + HIR after 60 minutes of liver ischemia and 24 hours of reperfusion. (**e**,**f**) Representative western blots and quantitative evaluation of p-p70S6K expression in kidney tissues from the rats subjected to control-operated, HIR, and Oct. + HIR after 60 minutes of liver ischemia and 24 hours of reperfusion. Ten rats were included in each study group. The results are presented as mean ± S.D. *P < 0.05 HIR vs. control group, ^#^P < 0.05 Oct. + HIR νs. HIR group. AKI, acute kidney injury; CTR, control; HIR, hepatic ischemia-reperfusion; Oct. + HIR, octreotide pretreatment and hepatic ischemia-reperfusion and Oct. + HIR+3MA, octreotide and 3-methyladenine pretreatment and hepatic ischemia-reperfusion.

The Akt/mTOR**/**p70S6K signaling pathways are essential for the regulation of autophagy. Akt, mTOR and p70S6K phosphorylation activates the signaling pathways and subsequent inhibition of autophagy^17^. To explore whether octreotide could affect the Akt/mTOR**/**p70S6K signaling pathways in the kidney after hepatic IR, we evaluated the phosphorylation of Akt/mTOR**/**p70S6K by western blot analysis. As shown in Fig. 5a–f, the phosphorylation of Akt/mTOR**/**p70S6K significantly increased in the kidney after HIR. Reduced phosphorylation levels were obtained after octreotide treatment (Oct. + HIR) compared to the HIR group. This data suggests that autophagy induced by octreotide treatment, may occur in part through the inhibition of the Akt/mTOR**/**p70S6K signaling pathway.

## Discussion

In this study, we demonstrated for the first time that octreotide reduces renal inflammation, apoptosis and protects against AKI after HIR. Moreover, octreotide was also noted to induce autophagy in the kidney after HIR, supporting its protective effects. Autophagy inhibition with 3MA markedly reversed the renoprotective, anti-inflammatory and anti-apoptotic effects of octreotide after HIR. Additionally, octreotide abrogated the activation of the Akt/mTOR**/**p70S6K signaling pathway in the kidney after HIR. These results support the idea that octreotide reduces AKI after HIR via its induction of autophagy. Further, the increasing degree of autophagy seems to be linked to the octreotide mediated Akt/mTOR**/**p70S6K pathway deactivation and reduction of kidney inflammation and apoptosis after HIR.

HIR is a frequent cause of AKI during the perioperative period, and the systemic inflammatory response triggered by liver cell death, associative oxidative stress, and the release of many tissue damage factors have been identified as key elements driving the pathophysiology of AKI^23^. AKI caused by HIR, is characterized by early renal endothelial cell death with subsequent renal inflammation and proximal tubular necrosis^24^. Furthermore, initiation of AKI after HIR further exacerbates liver injury creating a vicious cycle, which makes therapeutic interventions extremely difficult^25^. In our previous studies, we demonstrated that octreotide could decrease the serum levels of proinflammatory cytokines (e.g., TNF-α and IL-1β) and inhibit hepatocellular apoptosis, as well as protect the liver against ischemic/reperfusion injury^11^. When we further explored the role of octreotide on AKI after HIR in this study, we found that octreotide preconditioning could preserve the renal function and reduce injury after HIR. Moreover, octreotide could reduce TNFα, IL-6, MCP-1, and MIP-2 expression in the kidney after HIR. In addition, octreotide suppressed apoptosis in the kidney after HIR. Taken together, our previous and current findings imply that octreotide possesses anti-inflammatory and anti-apoptotic activity, all of which provide powerful protection against liver and kidney injury after HIR.

Similar to our studies, previous studies have also shown that octreotide exerts organ protective effects^8^,^9^,^10^. However, the underlying mechanism was largely unknown. This current study supports the idea that enhancing autophagy from octreotide may be a key protective mechanism in reducing AKI after HIR. Several lines of evidence support this concept. Firstly, octreotide induces autophagy in renal endothelial cells after HIR in the rat model. Secondly, inhibition of autophagy by 3MA reduced the anti-apoptotic effect of octreotide during AKI after HIR. Lastly, 3MA diminished the inhibitory action of octreotide on TNFα, IL-6, MCP-1, and MIP-2 expression in the kidney after HIR. These results imply that octreotide protects against kidney injury after HIR by enhancing autophagy.

As mentioned above, we demonstrated that octreotide suppresses apoptosis in the kidney after HIR. However, octreotide has been noted to induce apoptosis in different cancer cell lines *in vitro*^26^,^27^. This discrepancy may be due to different cell types, treatment factors, and experimental conditions etc. Moreover, octreotide mediated anti-apoptosis may mainly be due to the autophagy inducing effects of the agent. Indeed, there are profound interactions between autophagy and apoptosis: apoptosis is inhibited when autophagy is activated, whereas inhibition of autophagy can promote cell death and the activity of caspase proteins^28^,^29^.

Recent evidence supports the view that enhancing autophagy may be a novel approach to improve renal injury^22^. The mTOR/p70S6K pathway is the major inhibitory signal that shuts off autophagy and mTOR is a key kinase downstream of Akt. Activation of the Akt may result in mTOR/p70S6K pathway activation and subsequent autophagy inhibition^6^,^17^,^30^. Thus, modulation of the Akt/mTOR/p70S6K pathway may shed a potential therapeutic strategy in the pathogenesis of AKI. When we further explored the role of octreotide on the Akt/mTOR**/**p70S6K pathway, we found that octreotide inhibited the activation of Akt/mTOR**/**p70S6K, as shown by the reinforcing of the suppression of phosporylation of Akt, mTOR and p70S6K in the kidney after HIR. This indicates that octreotide induces autophagy, at least partially, mediated by deactivation of the Akt/mTOR**/**p70S6K pathway.

Interestingly, some previous studies have shown that activation of Akt signaling can protect against ischemia and reperfusion-induced kidney injury^31^,^32^. Thus, the activation of the Akt pathway may work as a double-edged sword; it plays a protective role against AKI, but in contrast, it also inhibits autophagy, which is another important protective mechanism against AKI. Overall, our findings demonstrated that although the Akt pathway was inhibited by octreotide, octreotide still showed a protective role against AKI.

This trial has limitations. The study was conducted in only one animal model and extrapolation from rats to humans is always difficult. Additionally, only one dose of octreotide was tested and it is unclear if there would be an enhanced effect with a larger dose.

In conclusion, our study demonstrated that octreotide inhibited kidney inflammation, apoptosis, and subsequently protected against kidney injury in the setting of HIR. This protective effect appears to be due to octreotide-induced autophagy, which is at least partially mediated by deactivation of the octreotide-stimulated Akt/mTOR/p70S6K pathway. These observations define a novel mechanism for octreotide in the control of AKI, and support the potential utility of octreotide to prevent and treat organ dysfunction after HIR.

## Materials and Methods

### Animals

All animal experimental protocols were according to the guide for the care and use of laboratory animals (8th edition) and all experimental protocol was approved by the Institutional Animal Care and Used Committees of Hunan Cancer Hospital, Changsha, China (Permit Number: 2015009). 40 male Sprague-Dawley rats weighing 200–250 g were purchased from the Center of Experimental Animals of Hunan Cancer Hospital (Changsha, China). Rats were housed in cages under controlled temperature (20–25 °C) and humidity (50–52%), with a standard daily light-dark cycle (light from 07:00 to 18:00). They were allowed free access to food and water.

### Model Establishment and Experimental Design

The animals were randomly divided into 4 groups (n = 10 each group); control group-surgery with no hepatic ischemia (CTR), hepatic reperfusion group with no intervention (HIR), hepatic reperfusion group given octreotide (Oct. + HIR) and hepatic reperfusion group given octreotide and 3-methyladenine (Oct. + HIR+3MA). All 3 animal groups with HIR were setup as described previously^33^. Briefly, the rats were placed under a heating lamp and on a 37 °C heating pad. After a midline laparotomy and intraperitoneal application of heparin 20 U, left lateral and median lobes of the liver were subjected to 60 min. of ischemia with a microaneurysm clip occluding the hepatic pedicle above the bifurcation. After 60 minutes, the liver was reperfused and the wound was closed. Twenty-four hours after reperfusion, the kidneys and plasma samples were collected for analysis. In the control group (CTR), rats had only the hepatic porta dissected without vascular occlusion. The rats in the octreotide and hepatic injury and reperfusion group (Oct. + HIR) received an injection of octreotide (A-Think Pharmaceutical Co., Jilin, China) 20 ug/kg intraperitoneally and 30 ug/kg subcutaneously 0.5 h before the onset of HIR; The octreotide, 3-methyladenine, and hepatic injury and reperfusion group (Oct. + HIR+3MA) were dosed with 3MA (30 mg/kg, ip) 0.5 h before the administration of octreotide and subsequent HIR as noted above.

### Assessment of renal function

Serum and urinary creatinine was measured using the modified Jaffe rate reaction in the clinical laboratory of The Hunan Cancer Hospital, Changsha, China. Urinary KIM-1 was measured with commercially available ELISA kits (R&D Systems Inc).

### Histological analysis

Kidney specimens were fixed in 10% buffered formalin, embedded in paraffin, stained with hematoxylin and eosin (H&E), and examined with a light microscope. Slides were reviewed blindly and scored with a semiquantitative scale. Higher scores represented more severe damage, with points given for the presence and extent of tubular epithelial cell flattening (1 point), brush border loss (1 point), cell membrane bleb formation (1 or 2 points), peritubular/proximal tubule leukocyte infiltration (1 point), interstitial edema (1 point), cytoplasmic vacuolization (1 point), cell necrosis (1 or 2 points), and tubular lumen obstruction (1 or 2 points).

### RNA Isolation and RT-PCR

Total RNA from the rat kidney tissue was extracted by using TRIzol reagent (Sigma, St. Louis, MO) in accordance with the manufacturer’s instructions. Real-time quantitative PCR, using SYBR Green detection chemistry, was performed on a Roche light Cycler Run 5.32 Real-Time PCR System. The primer sequences used were the following: TNF-α forward primer, 5′-TCTCAAAACTCGAGTGACAAGC-3′, reverse primer, 5′-GGTTGTCTTTGAGATCCATGC-3′; IL-6 forward primer, 5′- ATCTGCTCTGGTCTTCTGGA-3′, reverse primer, 5′-TGACCACAGTGAGGAAT

GTC-3′; MCP-1 forward primer, 5′-ACCTGCTGCTACTCATTCAC-3′, reverse primer, 5′-TTGAGGTGGTTGTGGAAAAG-3′; MIP-2 forward primer, 5′- CCAAGGGTTGACTTCAAGAAC-3′, reverse primer, 5′-AGCGAGGCACATCAGG

TACG-3′. Melt curve analyses of all real-time PCR products were performed and shown to produce a single DNA duplex. Quantitative measurements were determined by using the ΔΔ Ct method and expression of β-actin was used as the internal control.

### Immunohistochemical Staining

The prepared paraffin-embedded sections were dewaxed and rehydrated through a graded series of alcohol and then heated in an oven at 60 °C for 20 min. Antigens were recovered by incubating the samples in citrate buffer in a 95 °C water bath for 20 min, and then endogenous peroxidase was blocked by incubating them in 3% hydrogen peroxide for 20 min at 37 °C. Membranes were ruptured with 0.2% Triton X-100 at room temperature for 30 min and nonspecific binding sites were blocked with 5% bovine serum albumin at 37 °C for 20 min, followed by incubation at room temperature for 10 min. The kidney slices were then incubated overnight with antibodies directed against TNFα, IL-6, MCP-1, MIP-2, and a rabbit anti-rat antibody (Abcam, Cambridge, UK) at 4 °C. Antibody binding was analyzed with a diaminobenzidine kit. The slides were then counterstained with hematoxylin, dehydrated in graded ethanol and xylene, and mounted with Entellan (Merck, Darmstadt, Germany).

### Western Blotting

Kidney tissue proteins were extracted. Equal amount of total proteins (40 μg) were loaded onto 10% SDS-PAGE, fractionated by electrophoresis, and transferred to polyvinylidene fluoride (PVDF) membranes. Primary antibodies were used against cleaved caspase-3, Bcl-2p65, LC3, beclin-1, Akt, p-Akt, mTOR, p-mTOR, p70S6K and p-p70S6K (Cell Signal Technology, Boston, USA) and β-actin (Abcam, Cambridge, UK). Protein bands were visualized with ECL plus chemiluminescent substrate.

### Tunel Staining

Tunel staining was performed according to the instructions for the TUNEL assay kit (Roche, 11684795910). In each section, areas without significant necrosis in 10 different visual fields (200×) were analyzed for TUNEL-positive cells. A Tunel index was calculated by counting the total nuclei and the cells with brown nuclei in the peri-infarcted area of five visual fields. The TUNEL index was determined using the following formula: (number of stained cells/number of stained cells + number of unstained cells) ×100.

### Transmission electron microscopy

Kidneys were sectioned and photographed using a transmission electron microscope (HITACHI-7500 TEM, Japan) at 80 or 60 kV onto electron microscope film (Kodak, ESTAR thick base) and printed onto photographic paper. For quantification, 20 to 30 fields of low magnification (×1000) were randomly selected from each kidney, and digital images with scale bars were taken. Using Axio-Vision 4.0 software, the degree of autophagosomes per 100 μm 2 cytoplasmic area was evaluated.

### Statistical Analysis

Data are expressed as means ± S.D. Results were analyzed by one-way ANOVA and Student’s t test, using SPSS 16.0 software. Statistical significance was obtained when P values were less than 0.05.

## Additional Information

**How to cite this article**: Sun, H. *et al*. Octreotide Attenuates Acute Kidney Injury after Hepatic Ischemia and Reperfusion by Enhancing Autophagy. *Sci. Rep.* **7**, 42701; doi: 10.1038/srep42701 (2017).

**Publisher's note:** Springer Nature remains neutral with regard to jurisdictional claims in published maps and institutional affiliations.
